# Consumption in the Northwestern States

**Published:** 1878-08

**Authors:** H. A. Johnson

**Affiliations:** No. 4 Sixteenth St., Chicago


### Original Communications.

### CONSUMPTION IN THE NORTHWESTERN STATES.

By H. A. Johnson, M. D.

(*A paper read before the American Public Health Association, September*, 1877.)

I propose for discusssion several questions, to each of which I believe an appropriate answer at least is possible.

*First.* What is the relative frequency of phthisis pulmonalis in the Northwestern States, as compared with the Northeastern States of the Union ?

*Second.* Does phthisis pulmonalis, relatively to other causes of death, become more frequent as population increases in the United States ?

*Third.* What is the ratio of increase, if any, in the two sections of the country mentioned, namely, the North Atlantic States and the Northwestern States ?

*Fourth.* What are the causes that modify the prevalence of this disease in the two sections named, and more especially in the Northwestern States ?

In endeavoring to find an answer to the first question, namely, what is the relative frequency of phthisis in the Northwestern States, as compared with the Northeastern States, I have examined: 1st. The reports of the United States census for 1850, 1860 and 1870. 2nd. The reports of the Provost Marshal's office, giving the records of examinations and the causes for exemption from military duty of drafted men in the Northern States during our late civil war. 3rd. The records of the Chicago Board of Health and the sanitary history of Chicago for the last twenty years. 4th. Such other records, private and public, as have been accessible, bearing upon this subject.

As preliminary to the study, I have examined the general death-rate of the country, and of the two sections as determined by the census reports.

From the vital statistics of the United States census, 1850, 1860 and 1870, four classes of facts may be deduced:

1st. The increase or the decrease of the general death-rate of the whole country.

2nd. The increase or the decrease of the death-rate in the two sections which are the special subject of study.

3rd. The death-rate from phthisis in the whole United States and its increase or decrease.

4th. The death-rate from phthisis in the two divisions, as compared to the whole country and to each other, and the increase or decrease of this death-rate.

It is evident that the determination simply of the ratio of deaths from phthisis to all deaths may not give the true relative frequency of this disease as a cause of death—a greater or less mortality from other causes in a given population will modify the percentage of deaths from phthisis to the deaths from all causes, even though the deaths from phthisis may remain the same.

I find from the reports:

1st. That the general death-rate of the country from 1850 to 1870 has been diminished.

In 1850 the death-rate to population was 1.39; in 1860 it was 1.25; in 1870 it was 1.28 per cent. of the population. In 1850 epidemic cholera was present in many of the cities of the United States, and this fact should be considered in estimating the vitality of our population. I think, however, we may reasonably conclude that the general mortality in the United States has diminished during the twenty years.

I have also examined the problem in its relations to the two groups of States, namely, the New England States, New York and New Jersey, which I shall designate the New England or North Atlantic group; and Indiana, Michigan, Illinois, Wisconsin, Iowa and Minnesota, which I shall call the Northwestern group. I find that in both these groups the general death-rate has been diminishing from 1850 to 1870, but that in the Northwestern group the diminution has been greater than in the New

England group. In the Northwestern group it is as 106.1 for 1850 to 100 for 1870. In the New England group it is as 105.1 for 1850 to 100 for 1870, or, in other words, the improvement in the vital condition of the population of the Northwestern States is to that of the Northeastern as 106.1 to 105.1.

How do deaths from consumption compare with these conclusions as to the general death-rate in the whole country and in the two sections?

I find from the census reports:

1st. The deaths from consumption to 100,000 deaths from all causes in the whole United States were: in 1850, 10,376; in 1860, 12,453; in 1870, 14,199.

These figures indicate that phthisis in proportion to other causes of death is increasing in the United States, but they do not absolutely prove that this disease is really more prevalent or more fatal.

I find, however, that the deaths from consumption to 100,000 persons living was for the whole United States, 145.5 in 1850, 151.1 in 1860, 181.3 in 1870.

The proportion of deaths from phthisis to population, as well as to other causes of death, has therefore been increasing in the United States during the twenty years from 1850 to 1870, while, as already stated, the general death-rate of the country from all causes has been diminishing.

Let us examine more closely the valnable tables furnished by the U. S. government, with a view to an answer to the questions as to the death rate from phthisis in the northeastern and northwestern sections of the country, and the increase or decrease in that death rate.

The deaths reported from consumption in the North Atlantic States for 1850 were 251 to 100,000 persons living. In 1860, 225.5. In 1870, 253.75 for 100,000 persons living.

In the Northwestern States the deaths from consumption were in 1850, 111; in 1860, 124.5; in 1870, 134 per 100,000 persons living.

It appears, therefore, that while the general death-rate in both groups of States has been diminishing, the death-rate from consumption has increased in both, and that the increase has been, for the last twenty years, greater in the Northwestern States than in the North Atlantic States.

This increase in the ratio in the Northwestern States is limited however to the first decade, from 1850 to 1860. From 1850 to 1860 there was absolutely a decrease in the Northeastern group of States of 25.5 per 100,000 living persons, while in the Northwestern group there was an increase of 13.5 per 100,000 living persons. From 1860 to 1870, in the New England group, there was an increase of 28.25 per 100,000 living, and in the Northwestern group, an increase of only 9.5.

I think it quite probable that for 1850 the statistics in the newer Northwestern States were less accurate than in the North Atlantic group of States. Minnesota, for instance, in 1850, is credited with a population of 6,077 persons and only one death from consumption. Iowa, with nearly the same climatic conditions, had a population of 192,214 with 82 deaths from consumption per 100,000 persons living, while the general death rate was 111 per 100,000 living.

In 1860, the death-rate from consumption in the Northeastern States to that of the Northwestern States, was as 181 to 100. In 1870 the ratio was as 190 to 100. I think, therefore, we may reasonably conclude from these data—

1st. That in both groups the general movement of life is towards greater longevity.

2nd. That this movement is more active in the Northwestern States than in the Northeastern States.

3rd. That the death-rate from consumption, however, has increased in both groups.

4th. That for the twenty years the increase has been greater in the Northwestern than in the Northeastern group.

5th. That from 1860 to 1870 the reverse of this was true, the ratio of increase was greater in the Northeastern than in the Northwestern States.

Possible reasons for the decrease in the New England groups and greater increase in the Northwestern from 1850 to 1860, will be suggested when we come to consider the influences that modify the prevalence of the disease in the two sections.

If we examine the two groups of States more particularly, we find that in the Northeastern group consumption is more prevalent: 1st, along the sea-board; 2nd, along the shores of the lakes Erie and Ontario and the river St. Lawrence and along the Mohawk Valley. This general fact appears both from the census reports and from the Provost Marshal's report. In the Northwestern group the disease is more frequent along the eastern shore of Lake Michigan, the Ohio river and in the Valley of the Mississippi, including the Illinois, Rock, Wisconsin and western branches of the Mississippi, especially in the eastern part of Iowa.

In the Northeastern division we find the disease less prevalent west of the Connecticut river and in eastern and southern New York.

In the Northwestern division the most favored localities are along the western shore of Lake Michigan, and a belt extending southwest from Lake Michigan east of the Illinois down to the Mississippi above and below St. Louis, and the northwest part of Iowa and the whole of Minnesota.

The statistics of the Provost Marshal's office confirm the inference drawn from the census reports as to the general distribution of consumption.

The number of persons examined was half a million.

In the Northeastern States, New York and New Jersey, the ratio rejected on account of consumption per 1,000 examined, was 24.31. In the Northwestern group the ratio was 10.77 per 1,000. These figures deal only with a part of the population between 18 and 45 years of age, the period of life in which consumption is most likely to occur. So far as they indicate the distribution of the disease it seems to be more than twice as frequent in the Northeastern group as in the Northwestern group.

The statistics of the city of Chicago for the last twenty years bear upon the question of increase in the death rate from consumption in the northwest. In 1851 the death-rate from consumption was 12.4 per 10,000. In 1855, 20.2 per 10,000. In 1860, 25.3 per 10,000 persons living. In 1865, 19.5 per 10,000. In 1870, 16 per 10,000. In 1873, 16 per 10,000.

The ratio of deaths from consumption to the population from 1855 to 1873 has not therefore increased, but has decreased.

The proportion of deaths from consumption to all deaths for 22 years from 1852 to 1873 inclusive is 8.8 per cent. During the four years, 1870, 1871, 1872 and 1873, the ratio was 7.05 per cent. During the four years from 1855 to 1858 inclusive, the ratio of deaths from consumption to all deaths was 13 per cent.

During this period the ratio to population was also greater than during preceding years or subsequent to 1860. In 1852, 1854 and 1867, cholera was epidemic in Chicago, and the death-rate of consumption to all deaths was thereby diminished. Excluding these years and the death-rate of consumption to all deaths is 9.12 per cent. for the last four years of the series 7.05 per cent.

The inference from the statistics of this city are substantially in accord with those drawn from reports of the U. S. census. From 1850 to 1860 the death-rate of consumption, both to other causes of death and to the population, quite rapidly increased. From 1860 to 1870 the death-rate from this disease diminished both relatively and absolutely. In the whole Northwestern States there was, it is true, an increase from 1860 to 1870, but this increase was small as compared with that of the preceding decade.

What are the influences modifying the prevalence of consumption in these two groups of States?

1st. Physical condition and climate.

(a.) Altitude.

(b.) Temperature.

(c.) Moisture.

(*a.*) Rainfall. (*b.*) Atmospheric humidity. (*c.*) Soil.

2nd. Origin, density, occupation and modes of living of the people.

3rd. The general prosperity, conditions of comfort, and that best of all tonics, *hope.*

I cannot, in the time allotted me, attempt to study in detail the value of these different factors as modifying the prevalence of phthisis in the Northeastern and Northwestern States.

I must content myself with indicating a few only of the most important facts.

1st. If we compare the hypsometric determinations of the Northeastern and the Northwestern States, we find, with the exception of the sparsely inhabited higher ridges of the mountain chains in the Northern Atlantic group, but little difference in the general altitude of the two divisions, and the prevalence of phthisis seems to be modified, if at all, only to a very slight extent by this fact of elevation. We speak now only of the very moderate elevations at or below 1,000 feet. I am strongly inclined to think, however, that as a rule the higher elevations are more favorable for consumptives than the low districts, but probably, in part at least, for reasons independent of barometric pressure.

The difference in the mean temperature is also slight, and is equally inadequate as an explanation of the difference in the prevalence of this disease in the two sections. The frequency of storms, however, is greater in the Northeastern than in the Northwestern States, and it seems almost necessarily true that the changes of temperature are more frequent in the North Atlantic than in the Northwestern States.

3rd. The rainfall is greater in the New England group than in the Northwestern group. This is especially true for portions east of the White Mountains, and following the sea-coast to New Jersey.

In the Northwestern group there is more rainfall on the eastern shore of Lake Michigan than on the western shore, in the river valley than over the prairies. In other words, with only a few local exceptions, the prevalence of this disease is, in the same latitude, greater in regions having the greater rainfall and the most frequent changes of atmospheric humidity. The local exceptions, I think, may be explained by the geological formations. As a rule, other conditions being equal, the disease is less prevalent in regions where the soil is sand or gravel or made up of porous rocks than in hard impervious clays or primitive rocks. Gravel, sand or limestone admit of percolations through the surface layer, and a less amount of water is carried back into the air by evaporation or retained in the surface deposits. Moisture of the ground seems to have a marked influence in the production of consumption, as has been demonstrated, I think, by Drs. Bowdwich, Buchanan and others. If we go still further west, we find that upon the great plains and the interior plateaux, the rainfall, the humidity of the air and the soil is still less, and consumption becomes a rare disease, except as it is imported from regions of greater moisture, both of air and soil. I think we may justly believe that the diminished prevalence of the disease as a cause of death in Chicago during the last few years has been due, at least in part, to the great improvement in the drainage of the city and the surrounding country. In 1850 the city was low, with nothing but surface drainage, and the surrounding country was a marsh.

A second group of causes may be found in the origin, density, occupations, modes of living, etc., of the population. The population of the Northwestern States has been derived mostly from other parts of the United States and from foreign countries. Up to 1860 there were comparatively few persons of adult years born in these States. There has been a very large immigration from the North Atlantic States and from parts of Europe where consumption as a cause of death is much more prevalent than in this district. The proportion of immigrants was greater from 1850 to 1860 than from 1860 to 1870. The ratio of increase of this disease in the Northwestern States was greater during the former period than during the latter. While in the North Atlantic States there was actually a decrease of deaths from consumption during the former period and a large increase during the latter.

The facts were that many whole families, as well as a large number of individuals with consumptive tendencies, came to this Northwestern country, hoping thereby to prevent the development of the disease. In many cases this was successful. In many others it was not. There can be scarcely a doubt but that the mortality from consumption in the Northwestern States was increased, and in the New England group of States was diminished by the fact of such removal from one section to the other.

From an examination of my own records of consumption in private practice, I find that of 500 cases seen and examined in the last few years, 34 per cent. only were born in or had lived for any considerable time in the Northwestern States. These cases came from Chicago and a territory of one or two hundred miles around the city. Of my hospital cases I have not made an examination, but I think I am quite safe in saying that not more than 15 per cent. have been natives or have lived for any great number of years in the Northwestern States.

2nd. The population is less dense in the Northwestern States. A large proportion are engaged in agricultural pursuits. Less dust, fewer deleterious gases (except in Chicago), purer and dryer air, coming over the broad sweep of the prairies from the the mountains.

3d. Habits or modes of living also have an important bearing on the problem. We have fewer crowded, steam-heated workshops and factories. Our people also in their houses have better ventilation than the inhabitants of colder and moist districts. It is probably true that steam-heaters and close coal stoves have a positively deleterious influence upon the vitality of the population. In the earlier history of the Northwestern States these appliances of modern life were scarcely known. The open fire-place was found in every house and cabin, and a blazing wood fire carried up the capacious chimney all gases and odors generated in the dwelling, and drew into the living rooms an abundance of clean fresh air.

In the language of Moliere's *Médicin malgré lui*, "*Nous avons changé tout cela*," and I very much fear that the change has not been for the better.

The physical and social conditions of the people in almost all new countries are more favorable to health than in older and more densely populated districts. There are fewer persons sub ject to absolute want or privation. The very fact of immigrating to new lands implies hope and courage, and these are prophylactics and antidotes to disease, especially to consumption. As illustrating the effect of mental distress upon health, I venture to refer to one fact in the statistics of our own city after the great fire of 1871. During the year of 1871 there were in the city 6,975 deaths, and the proportion of deaths from consumption to all deaths was 7.8 per cent. During the year 1872 there were 10,156 deaths, and the proportion of consumption to all deaths was 7.09 per cent. One hundred thousand of our people were made homeless by that calamity. During the 14 months following the fire, 89,724 persons were treated by the physicians of the Relief and Aid Society. For the year 1872 the proportion of deaths from consumption among the fire patients to all deaths among the same patients was 23 per cent., while the proportion of deaths from consumption in the whole city to all deaths was only 7.09.

I think this fact illustrates in a striking manner the influence of mental distress in developing and hastening the progress of this disease.

There was no real want for food, clothing or shelter, but very many of these poor people had lost their all, often the hard earnings of many years, and to these there seemed to be no future but death. With hope and courage gone, they fell easy victims to the scourge that decimates annually 3,000,000 of human beings.

Finally, I beg to present what seem to me to be legitimate conclusions from this study of the movement of life from 1850 to 1875.

1st. The general death-rate is diminishing in the whole United States.

2nd. The death-rate is diminishing both in the Northwestern and Northeastern groups of States.

3rd. The diminution of the death-rate has been greater in the Northwestern than the Northeastern group.

4th. The death-rate from consumption to population has been increasing in the whole United States.

5th. In the Northeastern States from 1850 to 1860 there was a marked decrease, and from 1860 to 1870 a very decided increase to the death-rate from consumption.

6th. In the Northwestern States from 1850 to 1860 there was a very decided increase, and from 1860 to 1870 a very small increase in the death-rate from consumption.

7th. From the reports of the three periods, 1850, 1860 and 1870, it appears that the increase in the death-rate from consumption for the whole period was greater for the Northwestern than the Northeastern States, while the absolute death-rate from this disease in the Northwestern States was still only a little more than one-half of that in the North-Atlantic group of States.

Of the influences that modify the prevalence of the disease in these two sections, it seems probable that in the order of importance should be considered :

1st. The origin of the population.

2nd. The rain-fall, storm-movements, changes of the atmospheric temperature and humidity and character of soil as modifying its moisture.

3rd. Density, occupations and modes of living of inhabitants.

4th. Social and moral conditions—or, in other words, general well-being and prosperity.

This paper has reference to the facts of distribution, and the reasons for difference of distribution of consumption. It is only suggestive of modes of prevention in so far as it indicated probable influences of causation.

With the improvements in sanitary science and the encouragement to sanitary study furnished by such bodies as the American Public Health Association, and the interest manifested by the people in health problems, I trust we may find some means not only to prevent the increase but to diminish, if we may not eradicate this disease as a factor of mortality.

No. 4 Sixteenth St., Chicago.

